# Pregnancy Augments G Protein Estrogen Receptor (GPER) Induced Vasodilation in Rat Uterine Arteries via the Nitric Oxide - cGMP Signaling Pathway

**DOI:** 10.1371/journal.pone.0141997

**Published:** 2015-11-04

**Authors:** Teresa Tropea, Ernestina Marianna De Francesco, Damiano Rigiracciolo, Marcello Maggiolini, Mark Wareing, George Osol, Maurizio Mandalà

**Affiliations:** 1 Department of Biology, Ecology and Earth Sciences, University of Calabria, Rende, Italy; 2 Department of Pharmacy, Health and Nutritional Sciences, University of Calabria, Rende, Italy; 3 Maternal and Fetal Health Research Centre, The University of Manchester, Manchester, United Kingdom; 4 Department of Obstetrics, Gynecology and Reproductive Sciences, University of Vermont, Burlington, Vermont, United States of America; University of Southampton, UNITED KINGDOM

## Abstract

**Background:**

The regulation of vascular tone in the uterine circulation is a key determinant of appropriate uteroplacental blood perfusion and successful pregnancy outcome. Estrogens, which increase in the maternal circulation throughout pregnancy, can exert acute vasodilatory actions. Recently a third estrogen receptor named GPER (G protein-coupled estrogen receptor) was identified and, although several studies have shown vasodilatory effects in several vascular beds, nothing is known about its role in the uterine vasculature.

**Aim:**

The aim of this study was to determine the function of GPER in uterine arteries mainly during pregnancy. Uterine arteries were isolated from nonpregnant and pregnant rats.

**Methods:**

Vessels were contracted with phenylephrine and then incubated with incremental doses (10^−12^–10^−5^ M) of the selective GPER agonist G1.

**Results:**

G1 induced a dose-dependent vasodilation which was: 1) significantly increased in pregnancy, 2) endothelium-dependent, 3) primarily mediated by NO/cGMP pathway and 4) unaffected by BK_ca_ channel inhibition.

**Conclusion:**

This is the first study to show the potential importance of GPER signaling in reducing uterine vascular tone during pregnancy. GPER may therefore play a previously unrecognized role in the regulation of uteroplacental blood flow and normal fetus growth.

## Introduction

During pregnancy, uteroplacental blood flow increases significantly to allow the normal growth of the fetus. Reduced blood flow to the uteroplacental unit is observed in gestational diseases such as fetal growth restriction and preeclampsia, with serious consequences for pregnancy outcome. Estrogens may modulate uteroplacental vascular function since its plasma concentrations increase significantly during pregnancy, and an effect on vascular tone has been documented in many experimental and clinical contexts [1]. Estrogens act on the vasculature via three different receptors: the two classical nuclear estrogen receptors, ERα and ERβ, function traditionally as ligand-activated nuclear transcription factors [2], while a third membrane estrogen receptor termed G-protein coupled estrogen receptor (GPER, formerly GPR30) was recently identified as an orphan 7-transmembrane G protein-coupled receptor [3–7]. In the last decade, several studies have shown that GPER [8,9] mediates the action of estrogens and estrogen-like compounds in diverse pathophysiological conditions [10–15]. In addition, using the specific GPER agonists and antagonists namely G1 [16] and G15 [17], respectively, several studies have shown that GPER plays a role in the nervous, immune, reproductive and vascular systems [18]. The potential vascular relevance of GPER function was first observed in human vascular endothelial cells, in which flow (shear stress) induced its expression [7]. GPER is also expressed in both endothelial and smooth muscle cells throughout the cardiovascular system [19–21]. Although several vessel types have been assessed [22–24], GPER has not been investigated in the uterine vasculature, which supplies blood flow to the uterus and placenta and plays a crucial role in providing sufficient blood for normal placental exchange [25].

In this study we ascertained that GPER is expressed in the uterine circulation, its activation triggers a vasoactive effect primarily through the NO-cGMP signaling system in uterine arteries and that its effects may be altered during pregnancy.

## Material and Methods

### Animals

All experiments were conducted in accordance with the European Guidelines for the care and use of laboratory animals (Directive 2010/63/EU) and were approved by the local ethical committee of the University of Calabria. Surgery was performed under anesthesia to minimize pain and suffering. Female Sprague-Dawley rats were purchased from Harlan Laboratories (Italy). All animals were housed under controlled conditions on a 12-hour light/dark cycle and provided commercial chow and tap water *ad libitum*. Experiments were performed on age-matched pregnant and non-pregnant animals at 12–15 weeks of age. Pregnant animals were obtained by placing a female in proestrus with a fertile male overnight; detection of spermatozoa using a vaginal smear on the following morning was used to confirm day 1 of pregnancy. Animals were euthanized with inhalation of Diethyl ether followed by decapitation, the uterus was removed and uterine arteries were dissected free from connective and adipose tissue for subsequent experimentation.

### Pressure myography

Radial uterine arteries were obtained from non pregnant (NP) and pregnant animals (P) at 14 days of gestational age, i.e. approximately one week before term. Arterial segments (1–2 mm long) were transferred to the chamber of a small-vessel arteriograph. One end of the vessel was tied onto a glass cannula and flushed of any luminal contents by increasing the pressure before securing the distal end onto a second cannula using a servo-null pressure system (Living Systems Instrumentation). All vessels were continuously superfused with HEPES-physiological saline solution (HEPES-PSS) at 37°C, pressurized to 50 mmHg, and equilibrated for 45 min before beginning experimentation. Lumen diameter was measured by trans-illuminating each vessel segment and using a video dimension analyzer (Living Systems Instrumentation) in conjunction with data-acquisition software (Ionoptix) to continuously record lumen diameter.

Following equilibration, all vessels were pre-constricted with phenylephrine (0.1–1μM) to produce a 40–50% reduction in baseline diameter [26]. Once constriction was achieved and stable for about 10 minutes, the specific agonist of GPER, 1-(4-(-6-Bromobenzol(1,3)diodo-5-yl)3a,4,5,9b-tetrahidro-3Hcyclopenta(c-)quinolin-8yl)ethanone (G-1), dissolved in DMSO to prepare a stock solution of 1 mg/ml was added at a concentration of 10^−12^ ÷10^−6^ M. In some arteries the endothelium was removed (denuded artery) mechanically by hair and air perfusion, and the effectiveness of denudation confirmed by the lack of dilation to acetylcholine (10^−5^ M). Additional pharmacological experiments were carried out using the following inhibitors: 1) (3aS*,4R*,9bR*)-4-(6-Bromo-1,3-benzodioxol-5-yl)-3a,4,5,9b-3*H*-cyclopenta[*c*]quinoline (G-15, 10^-5^M) a specific antagonist of GPER; 2) N-nitro-L-arginine (L-NNA, 10^-4^M) + Nω-nitro-L-arginine methyl ester (L-NAME, 10^-4^M) for NOS and 3) ODQ (10^-5^M) for guanylate cyclase and 4) Paxilline (10^−5^ M) for BK_Ca_ channels. Vessels were pre-incubated with inhibitors for 20 minutes before pre-constriction with phenylephrine and addition of G1.

### Western blotting

Frozen uterine arteries from non pregnant and pregnant rats were powdered with a mortar and homogenized in 50 mM Hepes solution, pH 7.4, containing 1% (v/v) Triton X-100, 4 mM EDTA, 1 mM sodium fluoride, 0.1 mM sodium orthovanadate, 2 mM PMSF, 10 mg/ml leupeptin and 10 mg/ml aprotinin. In order to increase the amount of tissue for accurate measurements, uterine arteries from two rats were pooled. Homogenates were centrifuged at 13,000 rpm for 10 min and protein concentrations in the supernatant were determined according to the Bradford assay. Tissue lysates (40 μg of protein) were electrophoresed through a reducing SDS/10% (w/v) polyacrylamide gel and electroblotted onto a nitrocellulose membrane. After the transfer, the membranes were stained with Red Poinceau to confirm equal loading and transfer. Membranes were blocked and incubated with primary polyclonal IgG antibody GPER (N-15), β-tubulin (H-235-2) and appropriate secondary HRP-conjugated antibodies, all purchased from Santa Cruz Biotechnology (DBA, Milan, Italy). The levels of proteins were detected with horseradish peroxidase-linked secondary antibodies, and revealed using the Enhanced Chemiluminescence system (GE Healthcare, Milan, Italy).

### Drugs and Solutions

The HEPES-PSS contained the following (in mmol/L): sodium chloride 141.8, potassium chloride 4.7, magnesium sulfate 1.7, calcium chloride 2.8, potassium phosphate 1.2, HEPES 10.0, EDTA 0.5, and dextrose 5.0. All drugs tested were administered from stock solutions prepared daily, except for G1 and G15 where the stock solutions were frozen in small aliquots. G1 and G-15 were purchased from TOCRIS, distributed by R&D Systems (Milano, Italy), all the other chemicals were purchased from Sigma-Aldrich, Fisher Scientific, Cayman Chemical Co. unless otherwise specified.

### Statistical analysis

Vasodilation to G1was expressed as percent of maximally-relaxed diameter which was determined at the end of each experiment by the addition of a relaxing HEPES-PSS solution contining diltiazem (10 μM) + papaverine (100 μM). Data are expressed as means ± SEM, where n is the number of arterial segments studied. The n values refer to both number of vessels and number of animals. A normal distribution for all datasets was confirmed by Kolmogorov_Smirnov test, and differences in responses between groups were determined with two-way ANOVA for repeated measures analysis. Differences were considered significant at P ≤ 0.05.

## Results

**Fig 1:**
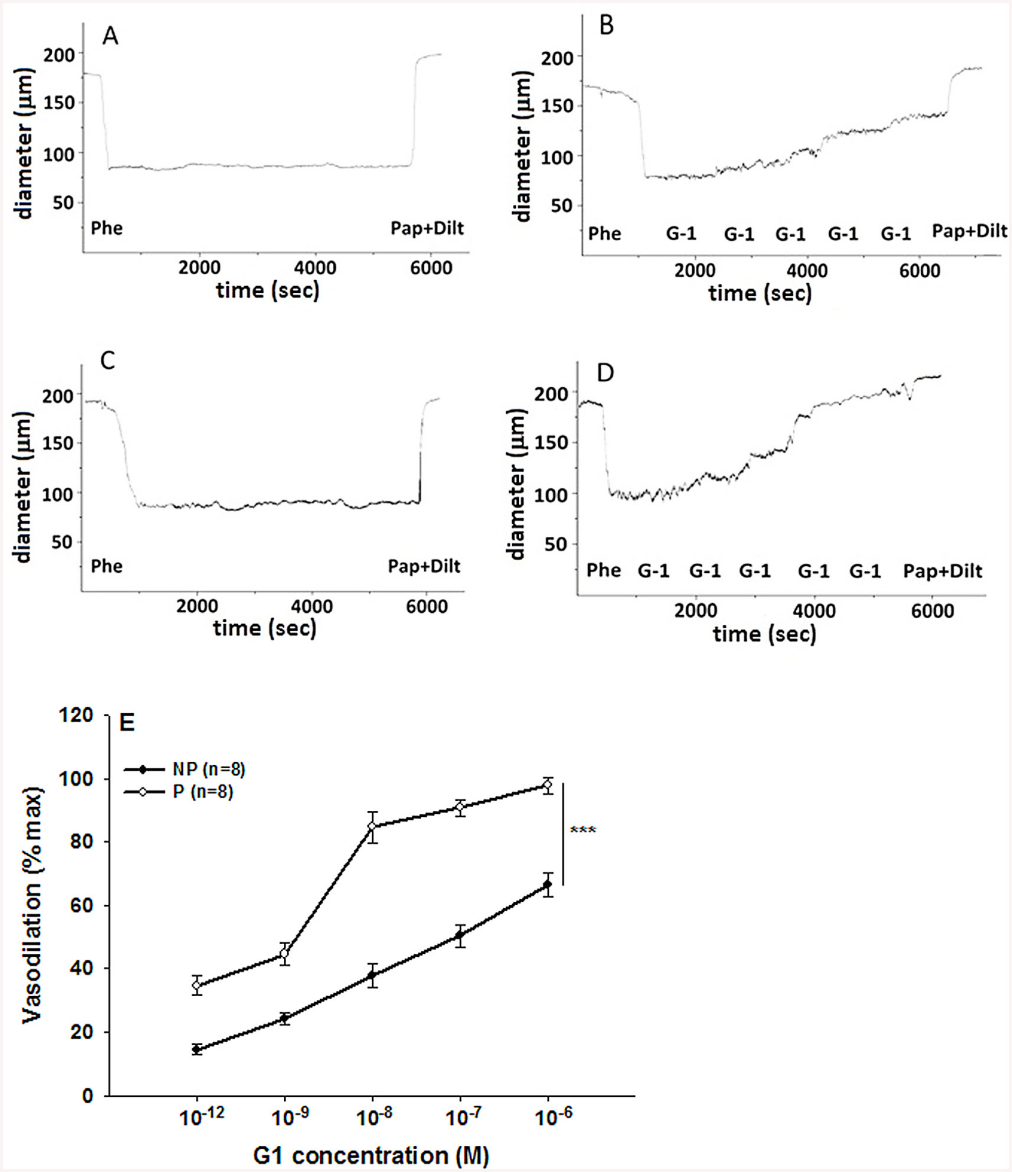
Concentration-response curves to G1 vasodilation in pressurized uterine radial arteries from non-pregnant (NP) vs. pregnant (P) rats. Uterine arteries were constricted with phenylephrine and then treated with the GPER agonist G1 at different concentration. An example of experimental records are showed in trace A and B for NP rat and in traces C and D for P rat. The Vasodilation of G1 was summarized in E and is expressed as a percentage of maximal relaxation (max) obtained in presence of papaverine and diltiazem. Data are reported as mean ± SEM; n indicates number of experiments. ***p <0.001.

**Fig 2:**
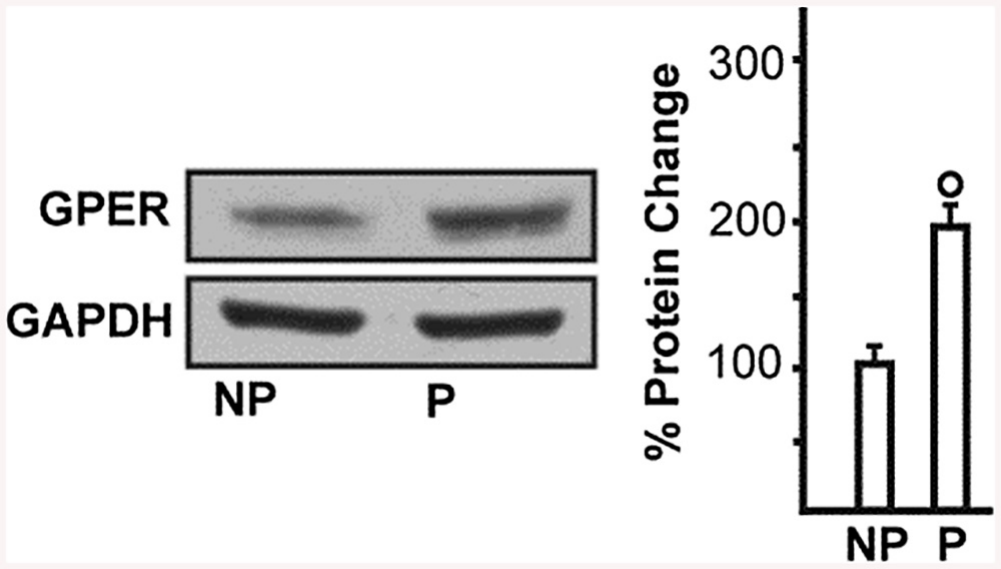
GPER expression in uterine radial arteries from nonpregnant and pregnant rats. Western blot showing. GPER protein expression in uterine arteries homogenates from non-pregnant (NP) and pregnant (P) rats. Side panel shows densitometric analysis of the blot normalized to β-tubulin. Percentage changes were evaluated as mean ± SEM of 3 experiments for each group. °p < 0.05 for the expression in P vs NP.

**Fig 3:**
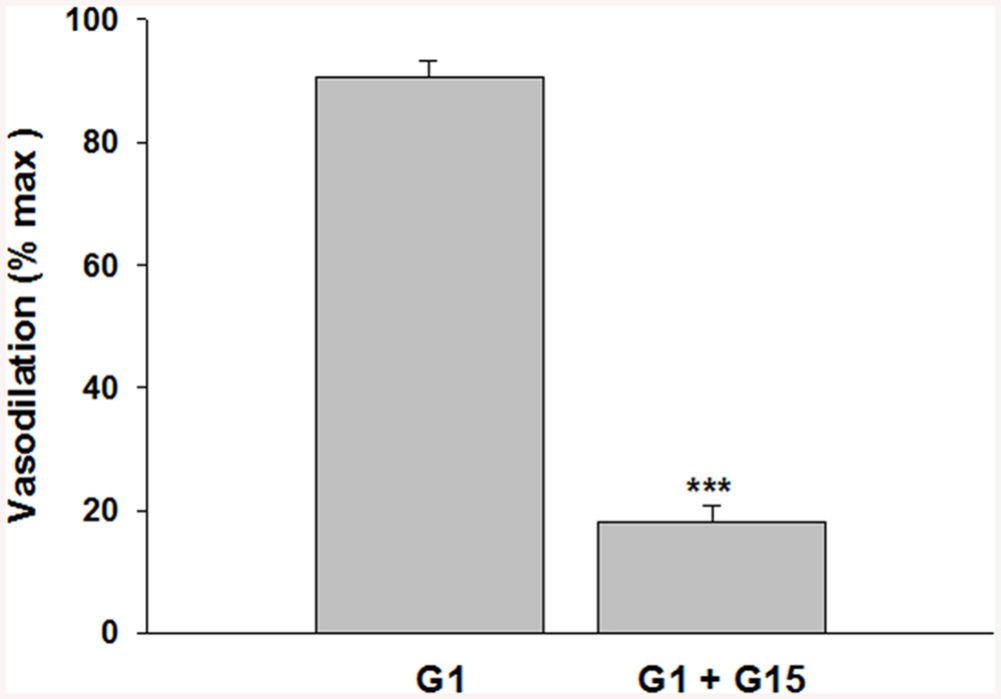
Effect of the specific GPER antagonist, G15, on G1 vasodilation in uterine radial arteries from pregnant rats. Inhibition of G1 (10^-7^M; n = 8) induced vasodilation of uterine arteries from pregnant rats by the GPER-specific antagonist G15 (10^-5^M, G1 ± G15, n = 5). Vasodilation is expressed as a percentage of maximal relaxation (% max) measured in a relaxing solution containing papaverine and diltiazem. Data are reported as mean ± SEM. ***p<0.001.

**Fig 4:**
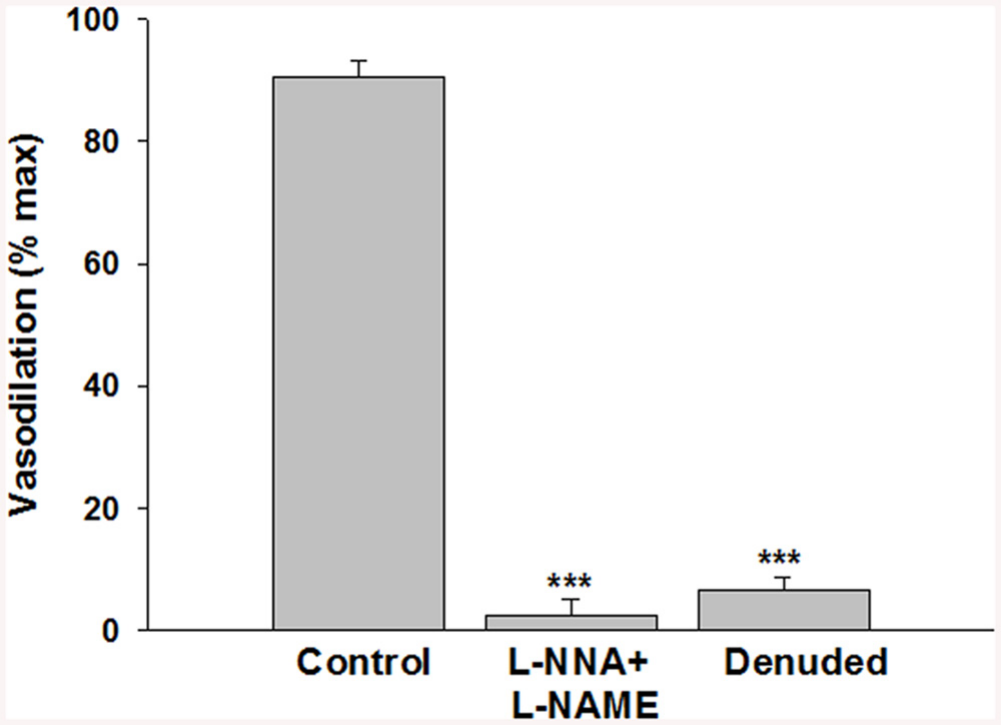
Effects of NOS inhibition and endothelial denudation on G1 vasodilation in uterine radial arteries from pregnant rats. G1 (10^−7^ M) was tested on intact radial uterine arteries in absence (Control, n = 8) vs. the presence of the nitric oxide synthase inhibition using a combination of L-NNA+L-NAME (n = 5). G1 was also tested on radial uterine arteries without endothelium (Denuded, n = 5). Vasodilation is expressed as a percentage of maximal relaxation (max) in papaverine and diltiazem. Data are reported as mean ± SEM. ***p<0.001.

**Fig 5:**
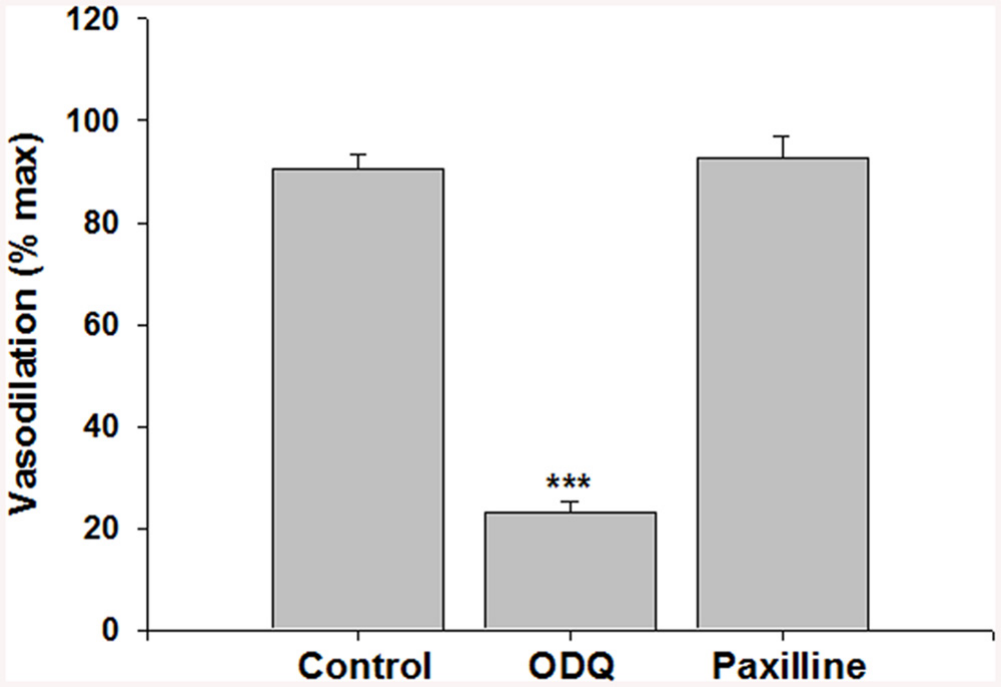
Effects of guanylate cyclase and BK_Ca_ channel inhibition on G1 vasodilation of rat uterine radial arteries. G1 (10^−7^ M) was tested on radial uterine arteries in absence (Control, n = 8) vs. presence of guanylate cyclase (ODQ, 10^-5^M, n = 5) or BK_Ca_ channel (paxilline 10^-5^M, n = 5) inhibition. Vasodilation is expressed as a percentage of maximal response (max) obtained in papaverine and diltiazem. Data are reported as mean ± SEM. ***p<0.001.

Phenylephrine is a potent vasoconstrictor of radial uterine arteries from NP and P rats as showing in the traces A and C respectively in Fig 1. Phenylephrine hold stable the constriction of the vessels for enough time necessary to observe clearly the action of the G1 showed in traces B and D respectively for NP and P. Several experiments were done and the data were summarized in Fig 1E that suggested G1, tested in the range (10^-12^–10^-6^M), induced vasodilation in a concentration-dependent manner in preconstricted radial uterine arteries from both NP and P rats. The vasodilation was significantly greater in vessels from P vs NP rats with a maximal efficacy of 97,8 ± 2,5% in P vs 66,5 ± 3,7% in NP; p<0.001 (Fig 1). Also GPER protein expression was significant higher in uterine artery from P rat vs NP with a p< 0.05 (Fig 2). The vasodilatory effect of G1 was almost entirely abolished in presence of the specific GPER antagonist, G15, as evidenced by an approximately 80% of reduction from 90,7 ± 2.6% (G1) to 18,2 ± 2.4% (G1 + G15), p<0.001(Fig 3). To understand the mechanism underlying the G1-induced vasodilation in radial uterine artery, several pharmacological experiments were carried out and the results shown that G1 vasodilation (90,7± 2.6%) was abolished by the inhibition of nitric oxide production (2,5±2,5%; p< 0.001) and also in denuded artery (6,6 ± 2,2%; p<0.001), Fig 4. Further, a significant reduction was observed by the inhibition of cGMP (23,1± 2,1%; p<0.001), while the inhibitor of BK channels (paxilline) did not affect the G1-induced vasodilation (Fig 5).

## Discussion

There were four principal findings in this study: 1) GPER activation with G1 induced significant vasodilation of rat uterine radial arteries; 2) G1 vasodilation was significantly augmented in pregnancy, 3) as was the expression of its protein in the arterial wall; 4) GPER vasodilation, which was effectively antagonized by the G15 inhibitor, was also endothelium-dependent and mediated by the NO-cGMP pathway without BK_Ca_ channel involvement.

During pregnancy, the maternal uterine circulation both vasodilates and undergoes a process of three-dimensional expansive remodeling [27]. Together, these processes result in a many-fold increase in uteroplacental blood flow that is requisite for normal fetal growth and myometrial function during parturition. Estrogen, whose concentrations increase progressively in the maternal circulation throughout pregnancy, has been reported to play a role in both processes of vasodilation [1,28] and remodeling [29]. In addition to ERα and ERβ, the classic nuclear estrogen receptors, a membrane G protein-coupled estrogen receptor termed GPER has recently been identified. There is increasing evidence that GPER is expressed in the cardiovascular system and may mediate some vascular estrogenic effects [30], although there have not been any studies to date on the uterine vasculature. The aim of this study was to evaluate the effects of GPER activation on uterine artery vascular tone, and to probe the underlying mechanisms by using the high affinity GPER-selective agonist (G1) and antagonist (G15). Neither compound shows any detectable activity towards the classical estrogen receptors [16,17]. Our study shows a potent vasodilator effect of G1-induced GPER activation in uterine resistance (radial) arteries. A similar vasorelaxant effect has been observed in several different types of arteries (cerebral, aorta, mesenteric, coronary, internal mammary), and in different species: rat, swine, human [22–24,31]. In human uterine arteries, there is one report of suggesting a lack of vasodilatory effect [32] but this may have to do with the use of U46619, a thromboxane receptor agonist, to contract the vessels since stimulus-specific effects have been reported previously; for example GPER agonists attenuated contractions to endothelin-1 but not serotonin [33]. Notably, we used Phe as the agonist in view of the rich adrenergic innervation of the uterine vasculature, and the fact that uterine vessels are more sensitive to catecholeamines than any other regional vascular bed [34]. We also found that GPER protein expression was increased significantly in uterine arteries from pregnant vs. non pregnant rats, an observation that may explain the greater magnitude of vasodilation.

G1-induced uterine artery vasodilation was endothelium dependent, and that this effect was mediated by NO since pharmacological inhibition of nitric oxide synthase virtually abolished the G1 vasodilation. Species differences may exist in this regard, however, since vessels from eNOS knockout mice were somewhat less reactive to G1 than wild type controls (data not shown), but a significant degree of relaxation nevertheless remained. This may reflect the existence of compensatory mechanism secondary to the loss of eNOS, e.g. upregulation of prostanoids or another endothelial vasodialtor. We also found that, while NO acted via the canonical cGMP pathway (based on the effectiveness of ODQ in blocking dilation), this effect did not involve BK_Ca_ channel activation. Activation of the NO-cGMP pathway following GPER stimulation was reported in several different types of arteries, e.g. mesenteric [24], cerebral [23], coronary [33] and carotid [20]. In denuded porcine coronary arteries, G1 did activate BK_Ca_ channels with consequent vasodilation that was not affected by the inhibition of nitric oxide synthase [35]. Thus, there may be both regional and species variations in post-receptor GPER coupling in endothelial and vascular smooth muscle cells.

In conclusion, this study is the first to show a GPER vasodilation in the uterine vasculature that is augmented in pregnancy,most likely secondary to upregulation of receptor expression, and involves endothelial NO release. Additional studies are warranted to determine whether GPER activation may offer a novel therapeutic mechanism for regulating uterine vascular tone and hemodynamics in gestational diseases associated with a reduction in uteroplacental blood flow such as preeclampsia and intrauterine growth restriction (IUGR).

## References

[1] Pastore MB, Jobe SO, Ramadoss J, Magness RR. Estrogen receptor-a and estrogen receptor-b in the uterine vascular endothelium during pregnancy: functional implications for regulating uterine blood flow. Semin Reprod Med. 2012;30:46–61. 10.1055/s-0031-1299597 22271294 PMC3674511

[2] Carroll JS, Brown M. Estrogen receptor target gene: an evolving concept. Mol Endocrinol. 2006; 20:1707–14. 16396959 10.1210/me.2005-0334

[3] Carmeci C, Thompson DA, Ring HZ, Francke U, Weigel RJ. Identification of a gene (GPR30) with homology to the G-protein-coupled receptor superfamily associated with estrogen receptor expression in breast cancer. Genomics 1997;45:607–17. 9367686 10.1006/geno.1997.4972

[4] Kvingedal AM, Smeland EB. A novel putative G-protein-coupled receptor expressed in lung, heart and lymphoid tissue. FEBS Lett. 1997;407:59–62. 9141481 10.1016/s0014-5793(97)00278-0

[5] O’Dowd BF, Nguyen T, Marchese A, Cheng R, Lynch KR, Heng HH, et al Discovery of three novel G-protein coupled receptor genes. Genomics 1998;47:310–3. 9479505 10.1006/geno.1998.5095

[6] Owman C, Blay P, Nilsson C, Lolait SJ. Cloning of human cDNA encoding a novel heptahelix receptor expressed in Burkitt’s lymphoma and widely distributed in brain and peripheral tissues. Biochem Biophys Res Commun.1996;228:285–92. 8920907 10.1006/bbrc.1996.1654

[7] Takada Y, Kato C, Kondo S, Korenaga R, Ando J. Cloning of cDNAs encoding G protein-coupled receptor expressed in human endothelial cells exposed to fluid shear stress. Biochem Biophys Res Commun. 1997;240:737–41. 9398636 10.1006/bbrc.1997.7734

[8] Thomas P, Pang Y, Filardo EJ, Dong J. Identity of an estrogen membrane receptor coupled to a G-protein in human breast cancer cells. Endocrinology 2005;146:624–32. 15539556 10.1210/en.2004-1064

[9] Revankar CM, Cimino DF, Sklar LA, Arterburn JB, Prossnitz ER. A transmembrane intracellular estrogen receptor mediates rapid cell signaling. Science 2005;307:1625–30. 15705806 10.1126/science.1106943

[10] Maggiolini M, Picard D. The unfolding stories of GPR30, a new membrane-bound estrogen receptor. J Endocrinol. 2010;204:105–114. 10.1677/JOE-09-0242 19767412

[11] De Francesco EM, Lappano R, Santolla MF, Marsico S, Caruso A, Maggiolini M. HIF-1α/GPER signaling mediates the expression of VEGF induced by hypoxia in breast cancer associated fibroblasts (CAFs). Breast Cancer Res. 2013;15:R64 23947803 10.1186/bcr3458 PMC3978922

[12] De Francesco EM, Pellegrino M, Santolla MF, Lappano R, Ricchio E, Abonante S, et al GPER mediates activation of HIF1α/VEGF signaling by estrogens. Cancer Res. 2014;74:4053–64. 10.1158/0008-5472.CAN-13-3590 24894716

[13] Lappano R, Rosano C, De Marco P, De Francesco EM, Pezzi V, et al Estriol acts as a GPR30 antagonist in estrogen receptor-negative breast cancer cells. Mol Cell Endocrinol. 2010;320:162–170. 10.1016/j.mce.2010.02.006 20138962

[14] Pupo M, Pisano A, Lappano R, Santolla MF, De Francesco EM, et al Bisphenol A Induces Gene Expression Changes and Proliferative Effects through GPER in Breast Cancer Cells and Cancer-Associated Fibroblasts. Environ Health Perspec. 2012;120:1177–1182. 10.1289/ehp.1104526 PMC3440081 22552965

[15] Albanito L, Lappano R, Madeo A, Chimento A, Prossnitz ER, Cappello AR, et al Effects of Atrazine on Estrogen Receptor α- and G Protein-Coupled Receptor 30-Mediated Signaling and Proliferation in Cancer Cells and Cancer-Associated Fibroblasts. Environ Health Perspect. 2015 (in press). 10.1289/ehp.1408586 PMC4421771 25616260

[16] Bologa CG, Revankar CM, Young SM., Edwards BS., Arterburn JB., Kiselyov AS, et al Virtual and biomolecular screening converge on a selective agonist for GPR30. Nat Chem Biol. 2006;2:207–12. 16520733 10.1038/nchembio775

[17] Dennis MK, Burai R, Ramesh C, Petrie WK, Alcon SN, Nayak TK, et al In vivo effects of a GPR30 antagonist. Nat Chem Biol. 2009;5(6):421–7. 10.1038/nchembio.168 19430488 PMC2864230

[18] Meyer MR, Prossnitz ER, Barton M. The G protein-coupled estrogen receptor GPER/GPR30 as a regulator of cardiovascular function. Vascul Pharmacol. 2011;55:17–25. 10.1016/j.vph.2011.06.003 21742056 PMC3216677

[19] Isensee J, Meoli L, Zazzu V, Nabzdyk C, Witt H, Soewarto D, et al Expression pattern of G protein-coupled receptor 30 in LacZ reporter mice. Endocrinology 2009;150:1722–30. 10.1210/en.2008-1488 19095739

[20] Broughton BRS, Miller AA, Sobey CG. Endothelium-dependent relaxation by G protein-coupled receptor 30 agonists in rat carotid arteries. Am J Physiol Heart Circ Physiol. 2010;298: H1055–H1061. 10.1152/ajpheart.00878.2009 20061543

[21] Lindsey SH, Carver KA, Prossnitz ER, Chappell MC. Vasodilation in response to the GPR30 agonist G-1 is not different from estradiol in the mRen2.Lewis female rat. J Cardiovasc Pharmacol. 2011; 5;57(5):598–603. 21326105 10.1097/FJC.0b013e3182135f1c PMC3095760

[22] Haas E, Bhattacharya I, Brailoiu E. Regulatory Role of G Protein-Coupled Estrogen Receptor for Vascular Function and Obesity. Circ Res. 2009;104(3):288–291. 10.1161/CIRCRESAHA.108.190892 19179659 PMC2782532

[23] Patkar S, Farr TD, Cooper E, Dowell FJ, Carswell HV. Differential vasoactive effects of oestrogen, oestrogen receptor agonists and selective oestrogen receptor modulators in rat middle cerebral artery. Neurosci Res. 2011;9;71(1):78–84. 10.1016/j.neures.2011.05.006 21624404

[24] Lindsey SH, Liu L, Chappell MC. Vasodilation by GPER in mesenteric arteries involves both endothelial nitric oxide and smooth muscle cAMP signaling. Steroids. 2014;81:99–102. 10.1016/j.steroids.2013.10.017 24246735 PMC3947732

[25] Mandala M, Osol G. Physiological remodeling of the maternal uterine circulation during Pregnancy. Basic Clin Pharmacol Toxicol. 2012;1;110(1):12–8. 10.1111/j.1742-7843.2011.00793.x 21902814

[26] Colton I, Mandalà M, Morton J, Davidge ST, Osol G. Influence of constriction, wall tension, smooth muscle activation and cellular deformation on rat resistance artery vasodilator reactivity. Cell Physiol Biochem. 2012;29(5–6):883–92. 10.1159/000178465 22613988

[27] Osol G, Mandala M. Maternal uterine vascular remodeling during pregnancy. Physiology 2009;24:58–71. 10.1152/physiol.00033.2008 19196652 PMC2760472

[28] Byers MJ, Zangl A, Phernetton TM, Lopez G, Chen DB, Magness RR. Endothelial vasodilator production by ovine uterine and systemic arteries: ovarian steroid and pregnancy control of ERalpha and ERbeta levels. J Physiol. 2005;5 15;565(Pt 1):85–99. 15774511 10.1113/jphysiol.2005.085753 PMC1464491

[29] Van der Heijden OW, Essers YP, Spaanderman ME, De Mey JG, van Eys GJ, Peeters LL. Uterine artery remodeling in pseudopregnancy is comparable to that in early pregnancy. Biol Reprod. 2005;73(6):1289–93. 16120827 10.1095/biolreprod.105.044438

[30] Haas E, Meyer MR, Schurr U, Bhattacharya I, Minotti R, Nguyen HH, et al Differential effects of 17β-Estradiol on function and expression of estrogen receptor α, estrogen receptor β, and GPR30 in arteries and veins of patients with atherosclerosis. Hypertension. 2007;49:1358–1363. 17452498 10.1161/HYPERTENSIONAHA.107.089995

[31] Arefin S, Simoncini T, Wieland R, Hammarqvist F, Spina S, Goglia L, et al Vasodilatory effects of the selective GPER agonist G-1 is maximal in arteries of postmenopausal women. Maturitas. 2014;78:123–30. 10.1016/j.maturitas.2014.04.002 24796498

[32] Corcoran JJ, Nicholson C, Sweeney M, Charnock JC, Robson SC, Westwood M, et al Human uterine and placental arterie sexhibit tissue-specific acute responses to 17b-estradiol and estrogen-receptors pecific agonists. Mol Hum Reprod. 2014;20(5):433–41. 10.1093/molehr/gat095 24356876 PMC4004081

[33] Meyer MR, Baretella O, Prossnitz ER, Barton M. Dilation of epicardial coronary arteries by the G protein-coupled estrogen receptor agonists G-1 and ICI 182,780. Pharmacology 2010;86:58–64. 10.1159/000315497 20639684 PMC3201835

[34] Magness RR, Rosenfeld CR. Systemic and uterine responses to alpha-adrenergic stimulation in pregnant and nonpregnant ewes. Am J Obstet Gynecol. 1986;155(4):897–904. 3766646 10.1016/s0002-9378(86)80047-3

[35] Yu X, Ma H, Barman SA, Liu AT, Sellers M, Stallone JN, et al Activation of G protein-coupled estrogen receptor induces endothelium independent relaxation of coronary artery smooth muscle. Am J Physiol Endocrinol Metab. 2011;301:E882–E888. 10.1152/ajpendo.00037.2011 21791623 PMC3213995

